# Conventional and Omics-Based Approaches to Investigate Sustainable Edible Coatings for Postharvest Preservation of Fruits and Vegetables

**DOI:** 10.3390/ijms27073014

**Published:** 2026-03-26

**Authors:** Tiziana Maria Sirangelo, Davide Barboni, Martina Catani, Natasha Damiana Spadafora

**Affiliations:** 1 Division Biotechnologies, ENEA—Italian National Agency for New Technologies, Energy and Sustainable Economic Development, Casaccia Research Center, Via Anguillarese 301, 00123 Rome, Italy; 2 Department of Chemical, Pharmaceutical and Agricultural Sciences, University of Ferrara, Via L. Borsari 46, 44121 Ferrara, Italy; brbdvd1@unife.it (D.B.);; 3 Department of Environmental and Prevention Sciences, University of Ferrara, Via L. Borsari 46, 44121 Ferrara, Italy

**Keywords:** edible coatings, postharvest, polysaccharides, proteins, lipids, metabolomics, transcriptomics

## Abstract

Edible coatings (ECs) derived from natural biopolymers represent an effective preservation strategy for fruits and vegetables and a promising postharvest approach aligned with the increasing demand for sustainable agricultural practices. These Generally Recognized As Safe (GRAS)-based coatings, which are mainly polysaccharide-, protein-, and lipid-based, can extend shelf-life with minimal impact on texture, flavor, and nutritional value, reducing reliance on synthetic packaging and helping mitigate food loss and waste. Beyond acting as a physical barrier, ECs can significantly influence fruit and vegetable metabolism by modulating biochemical and molecular processes. This review focuses on these effects by summarizing evidence from conventional analytical methods, including targeted metabolite analyses, as well as omics-based approaches, primarily transcriptomics and metabolomics, which remain poorly explored in the current EC research literature. Furthermore, integrated metabolomic and transcriptomic analyses are examined, as they offer a more comprehensive understanding of the molecular mechanisms underlying quality attributes, stress responses, and preservation outcomes. Collectively, this work offers detailed insights into coating-induced changes in metabolite profiles and gene expression in coated fruits and vegetables, including formulations derived from agri-food by-products and coatings enriched with bioactive compounds with antioxidant, antimicrobial, and antifungal properties. Overall, by addressing a current gap in the literature, it provides an integrative and innovative framework for interpreting coating performance at both applied and molecular levels, with potential relevance for the agri-food industry and for future research aimed at developing more sustainable, effective, and commodity-tailored postharvest technologies.

## 1. Introduction

Fruits and vegetables represent a major source of essential nutrients in modern society, as they provide a wide range of minerals, vitamins, and phytochemicals for human health [1]. Although it was demonstrated that minimal processing methods, such as washing, cutting, and modified atmosphere packaging (MAP), preserve the freshness of food products, the shelf-life of fruits and vegetables is still quite short [2]. As a result, approximately one-third of all food is wasted or lost, accounting for more than 1 billion tons per year [3,4]. In the case of fruits and vegetables, quality deterioration and postharvest losses are, to some extent, unavoidable, and several studies indicate that 25–50% of these products may become unsuitable for human consumption [5,6]. This further emphasizes the need for effective strategies to preserve their quality, improve postharvest management, and reduce waste.

Edible coatings (ECs) are made of food-grade ingredients, are safe for human consumption, and are processed to make thin, semi-solid films [7,8]. They are specifically designed to provide multiple functional benefits, such as preventing fruit decay throughout the whole storage and distribution chain. Moreover, ECs provide protection against physical damage, regulate vapor and fluid loss, and act as a barrier against both visible and UV light [9]. Because these effects complement atmosphere-based preservation strategies, ECs are often combined with MAP; however, the potential synergistic effects of this combination remain poorly understood, and further research is needed [10].

For EC formulation, it is essential to use compounds considered safe for consumption, classified as “Generally Recognized As Safe” (GRAS) by the Food and Drug Administration (FDA) in the United States, or by similar agencies in other countries [7,8]. In recent decades, numerous EC formulations incorporating diverse compounds have been developed, based on edible and biodegradable components derived from a range of natural sources with distinct structures and functions. These intrinsic features have positioned ECs as particularly attractive sustainable alternatives, and recent efforts have increasingly focused on developing effective and economically viable ECs to reduce reliance on conventional plastic packaging and minimize waste [11,12]. Accordingly, a major line of research has been the production of ECs from renewable biological ingredients, including biodegradable polymers from environmentally friendly sources such as algae, plants, and microorganisms. In parallel, EC development has progressively embraced circular-economy concepts, exploiting agri-food by-products and waste material, such as fruit and vegetable peels/leaves, as low-cost and readily available resources with substantial potential for postharvest quality preservation [13]. More broadly, this approach highlights the potential of ECs not only as postharvest preservation tools but also as platforms for biomass valorization, enabling the recovery of functional ingredients from agro-industrial residues and their reintegration into the food supply chain. In this way, EC development supports closed-loop and resource-efficient strategies by reducing waste generation, promoting more efficient use of biological resources, and facilitating the replacement of conventional fossil-based packaging materials [13,14].

Despite this progress, most evidence on EC performance still derives from endpoint quality traits and targeted measurements, with many studies [15,16] primarily relying on targeted biochemical assays to evaluate treatment-induced changes in specific metabolite classes, particularly compounds of nutritional interest or markers closely associated with ripening. These approaches provide only a partial view of the underlying metabolic changes induced by EC treatments; for this reason, omics-based approaches have gained increasing attention as powerful tools to provide a global view of biological processes. In this context, gene expression-based methods could provide valuable insights for studying the molecular mechanisms underlying fruit and vegetable ripening. In particular, transcriptomics may enable a deeper understanding of the mechanisms responsible for ripening delay upon EC treatment, facilitating the postharvest stage management. Moreover, this approach may provide valuable information for the identification of candidate genes directly involved in the response to EC application. At a downstream level, further insights can be obtained through metabolomics, which represents a powerful strategy to complement gene expression studies by enabling a comprehensive analysis of primary metabolism, essential for the qualitative characterization of fruits and vegetables during the postharvest stage [17]. Furthermore, metabolomics allows the investigation of several other metabolic pathways, including those associated with the maintenance of organoleptic properties during postharvest storage.

However, the growing omics literature has not yet been systematically integrated into existing EC reviews, which still predominantly frame coating performance through physicochemical traits and targeted assays. In particular, reviews specifically discussing and comparing conventional analytical approaches with transcriptomics, metabolomics, and multi-omics studies on EC-treated fruits and vegetables are still lacking. The present work aims to address this gap by providing a more mechanistic and integrative perspective on coating performance.

The present work first provides an overview of the composition and functional enhancement of edible coatings, the use of food by-products as materials for edible coatings, and the role of ECs in the postharvest preservation of fruits and vegetables. Evidence on polysaccharide-, protein-, and lipid-based ECs is also discussed, together with formulations incorporating bioactive compounds. The review then focuses on recent studies employing conventional analytical, biochemical, and targeted metabolite analyses, as well as omics-based approaches to elucidate the effects of EC treatments on fruits and vegetables. It also examines transcriptomic, metabolomic, and multi-omics reports on EC-treated horticultural products, linking molecular responses to postharvest quality outcomes. This approach highlights coating-responsive pathways and provides a framework to support more comprehensive and predictive EC design. Finally, it proposes a forward-looking strategy, based on the integration of conventional and omics-based methods, for studying coating effects at both applied and molecular levels, with potential relevance for the agri-food industry and for future research aimed at developing more sustainable, effective, and commodity-tailored postharvest technologies.

This work was conducted as a narrative review aimed at providing an updated and comprehensive overview of conventional and omics-based approaches used to investigate EC effects on fruits and vegetables. The literature search was carried out using major scientific databases, including Scopus, Web of Science, and PubMed, with combinations of keywords such as “edible coatings”, “postharvest”, “fruits”, “vegetables”, “targeted metabolite analysis”, “metabolomics”, “transcriptomics”, and “multi-omics”. References were selected to ensure relevance and scientific rigor, prioritizing studies published in peer-reviewed journals over the past ten years, with a particular emphasis on targeted metabolite analyses, metabolomics, and transcriptomics. In addition to recent publications, key and highly cited studies were included to highlight major research milestones in the field.

Overall, this review is intended to serve as a valuable resource for future research aimed at exploring molecular-level responses to EC treatments in horticultural products.

## 2. Composition and Functional Enhancement of Edible Coatings

The EC components are usually classified, according to their chemical nature, into polysaccharides, proteins, or lipid-based ECs.

Polysaccharides are relatively stable compounds and are commonly used to produce films around food products. These include starch, alginates (ALs), chitosans (CHs), pectins (PEs), cellulose, and gums [9]. Among them, starch has been successfully used as a coating for various fruits, such as strawberry [18] and pear [19], with promising results in shelf-life extension by preventing microbial contamination and increasing the antioxidant property [18,20]. ALs can be isolated from algae processed to produce translucent, shiny-looking coatings that have been shown to delay ripening and prevent fruit dehydration [21]. CH is the result of the deacetylation of chitin, which is considered the second most abundant biopolymer on earth after cellulose and can be easily obtained from the exoskeletons of insects, mollusks, crustaceans, and even fungi [22]. CH is also characterized by significant antimicrobial properties, inhibiting the growth of various types of fungi, yeasts, and bacteria [23]. These properties are attributed to the positive charges of the amino groups in CH, which interact electrostatically with negatively charged components on microbial membranes, thereby exerting antimicrobial activity [24]. Moreover, CH could reduce the respiration rate of fruits, delaying decay, by forming a coating and regulating the permeability of carbon dioxide and oxygen [25]. PE is a natural polysaccharide obtained from plants. Coatings made of PE have been proven to extend the shelf-life of fruits and vegetables [26] and to improve firmness and delay weight loss of fresh-cut fruits [27]. When formulating PE-based ECs, it should be considered that PE from different fruits may differ in their degree of esterification, acetylation, and amidation, which in turn affect their gelling behavior and functional performance in coating systems [28]. Among cellulose derivatives, the most commonly used in the food industry is carboxymethyl cellulose (CMC). It is GRAS for human health and possesses ideal properties for use in the preparation of coatings aimed at preserving the texture and organoleptic properties of fresh products [29]. The term “gum” generally refers to a broad class of polysaccharides capable of forming viscous aqueous solutions and stabilizing emulsions [30]. Many gums are of plant origin and may be derived from different parts of the plant, such as seeds, roots, and exudates, as well as from seaweed (such as AL and agar) [31]. Gum-based ECs have been successfully used in recent years to form a protective layer around food, extending the shelf-life and maintaining product quality over time [31].

In addition to the most established polysaccharides, emerging biopolymers are also attracting increasing interest for edible coating development. Among these, pullulan, a microbial polysaccharide characterized by good film-forming ability, transparency, and oxygen barrier properties, has shown promising potential for postharvest applications, including fruit preservation and shelf-life extension [32]. Likewise, fenugreek gum has recently gained attention as an innovative polysaccharide source because of its film-forming and stabilizing properties, particularly in composite formulations, and its potential use in sustainable coating systems [33]. Although these materials have been investigated less extensively than chitosan and other conventional polysaccharides, they reflect the ongoing innovation in biopolymer-based edible coatings and may represent valuable alternatives for future postharvest applications.

Proteins can also be used to create ECs and are obtained from either animal or plant sources. Animal-derived proteins include casein, collagen, gelatin, and whey, while plant-based proteins comprise gluten (mainly from wheat), zein (from corn), as well as other proteins from soy, peas, peanuts, rice bran, and various seeds. Among these, gelatin is the most widely used protein for the preparation of ECs and has been successfully applied as a coating for cherries [34] and other fresh foods, such as strawberries [35]. The hydrogen-bonded structure of protein-based coating provides a higher barrier capability compared to lipids and polysaccharide coatings [36], as well as conferring superior tensile strength when compared to CH-based ECs [37].

Lipids are quite effective in preventing water loss in food due to their low water affinity and can also enhance product quality and appearance. Commonly edible lipids used for EC formulations include beeswax, candelilla and carnauba wax, and several kinds of fatty acids [38]. Carnauba wax, a natural vegetable wax obtained from the leaves of Copernicia prunifera, is widely used in ECs due to its optimal film-forming and hydrophobic properties. Its beneficial effects include enhancing fruit surface gloss, delaying postharvest weight loss, contributing to the extension of shelf-life, and the maintenance of visual quality in coated food products. For instance, in Miranda et al. [39], a carnauba wax nanoemulsion based on GRAS ingredients was successfully used to improve the postharvest quality of tomatoes, confirming the previously mentioned beneficial effects. Beeswax has also been used to preserve several fruits during the postharvest stage. For instance, this coating treatment has shown the ability to delay the decline in weight loss, firmness, and to preserve lycopene in fresh tomatoes [40].

To further enhance the ECs’ function, coatings may be boosted by incorporating preservatives. For instance, bioactive compounds such as ascorbic acid (AA) and citric acid (CA), along with different types of essential oils, allow for a significant delay in food deterioration [41,42]. Furthermore, they can be useful not only to extend the shelf-life but also to obtain functional properties and relevant benefits for human health, thanks to the antioxidant, antimicrobial, antifungal, and probiotic properties of many of them [43].

Importantly, the effectiveness of these additives depends not only on their incorporation into the coating matrix but also on how uniformly the formulation is deposited on the product surface, since coating thickness and homogeneity influence barrier performance and the release of active compounds. From a practical standpoint, ECs can be applied using several techniques that aim to form a homogeneous layer of a given thickness around the food item. Dipping is the most commonly employed method. It consists of immersion of the food in the coating formulation, thereby generating a thin layer on the product [44]. Spraying is also widely applied, as the atomized droplets spread evenly over the surface, forming a uniform and homogeneous coating [44]. Panning represents another efficient and reliable method, in which food items spin in a large pan and are coated as they tumble. This technique, although originally developed for pharmaceutical applications, has proven to be particularly suitable for round-shaped food items [44].

However, regardless of the method, ECs usually exhibit limited barrier and mechanical properties; therefore, recent studies have focused on enhancing these properties by employing nanotechnology, multilayer coatings, or the incorporation of specific components [45]. Incorporating nanomaterials, such as nanoparticles and nanoemulsions, into the coating matrix has been shown to significantly improve key properties, including barrier function and controlled release of bioactive compounds [46,47]. Furthermore, challenges associated with traditional EC techniques, including off-flavors and uneven application, can be effectively mitigated through nanotechnology [46]. Multilayer ECs can be prepared by combining selected materials such as proteins (e.g., silk fibroin) and polysaccharides (e.g., CH, PE) in order to create coatings with improved mechanical strength, flexibility, and shelf-life extension for food products [48].

**Figure 1:**
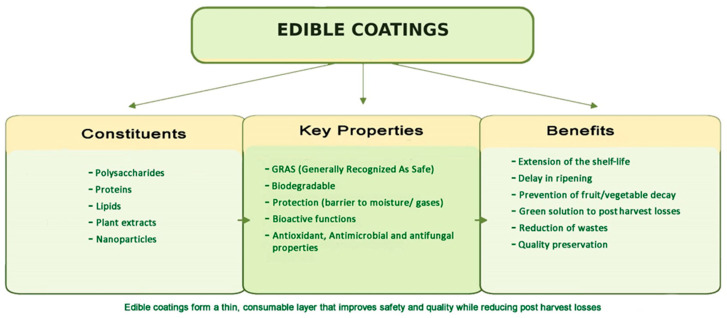
Major functionalities, properties, and benefits of edible coatings in fruit and vegetable preservation.

EC constituents, key properties, and related benefits are schematically summarized in Figure 1.

## 3. Food By-Products as Materials for Edible Coatings

Agri-food processing generates a substantial volume of by-products that can be valorized not only for energy recovery but also for packaging applications.

Plant-derived by-products, including seed kernels, peels, and leaves, often contain high levels of biopolymers such as cellulose, PEs, starch, and proteins, which are key components for EC formulation [49]. Furthermore, polyphenols present in peels, roots, seeds, and skins have been shown to inhibit lipid oxidation and bacterial growth in packaged foods [50].

Seed kernels of fruits are an important source of amylose and are one of the main components of starch. Their structure and biosynthetic characteristics have been carefully studied [51]. For instance, it has been demonstrated that the antioxidant components of avocado seed can help to prevent browning caused by polyphenol oxidase (PPO) [52]. Moreover, the effects of mango seed kernel starch-based active ECs enriched with lemongrass essential oil have been investigated, demonstrating the effectiveness of this coating in extending the shelf-life of guava fruit while maintaining its quality attributes [53].

Apple, mango, pomegranate, and pineapple peels have been reported to contain a high level of PEs. PE extracted from apple pomace has been employed to preserve fresh-cut apple quality [54]. PE from mango peels has been used as EC to extend the shelf-life of the same fresh fruit, proving its ability to delay ripening, decrease the total soluble solids (TSS), and soften the texture [55]. Pomegranate peel, which is rich in phenolic compounds, has been explored as a sustainable source of bioactive ingredients for the formulation of ECs with antioxidant and antimicrobial properties [50]. Pineapple peel, characterized by high water barrier and antioxidant properties, is often incorporated in commercial PE films [56]. Furthermore, several aromatic plants and fruit peels were used to produce EOs, often added into ECs for their bioactive properties [57].

Leaves represent a substantial fraction of organic waste, and several studies have focused on their use as potential substrates to obtain bioactive compounds. For instance, Zhang Y. et al. [58] obtained an active extract from loquat leaves and incorporated it into the formulation of an EC to extend the shelf-life of Nanfeng tangerines by preserving their postharvest quality. Aguilar-Veloz et al. [59] used leaf extracts rich in phenolic compounds as additives in the formulation of a PE-based EC with antifungal properties. This coating proved highly effective in controlling *Alternaria* sp. in tomatoes, one of the main pathogens responsible for significant production losses in this crop.

In addition, various residues from fruits, vegetables, and other natural sources have also been investigated and reviewed for their potential use in EC formulations [50,60]. For instance, the potential use of tomato waste materials, such as tomato branches, green fruit, and spoiled fruit, has been studied as a component of ECs [61], since these residues are rich in phenolic compounds as well as flavoring compounds. Fibers from mushroom by-products have also been investigated for use in ECs, demonstrating an improvement in mechanical strength and gas barrier properties [62]. Moreover, cellulose and lignin from food by-products and waste biomass [63] have been proposed in the packaging sector to strengthen biodegradable films, as recently highlighted by several authors [13,64].

Beyond their technological potential, this valorization pathway is also driven by sustainability and policy priorities. In this regard, the European Union strongly promotes research on food by-products and their valorization, encouraging the food industry to minimize waste generation [65]. This includes strategies aimed at recovering biopolymers from agro-industrial residues for subsequent incorporation into ECs and other bio-based materials [11,13].

## 4. Postharvest Quality Preservation of Fruits and Vegetables Using Edible Coatings

Ensuring the postharvest safety and quality of fruits and vegetables requires considering complex environmental factors in addition to the presence and proliferation of postharvest pathogens. Effective regulation of senescence, biotic stress resistance, and pathogen-driven diseases is critical for understanding the mechanisms underlying postharvest safety, quality, and yield [66].

Notably, postharvest deterioration is often accelerated by ripening-associated metabolic changes, including increased respiration and hormone-driven softening, which can rapidly compromise texture, flavor, and marketability. Many climacteric fruits (e.g., apples, bananas, tomatoes) are characterized by a respiratory burst that closely coincides with ethylene production levels peaks as the ripening stage starts [67]. These fruits are generally harvested when still at a premature stage, and a fine modulation of ethylene biosynthesis or signaling is a crucial factor for extending their shelf-life. In relation to this, Yamamoto et al. [68] reported that CH-based coatings significantly reduce ethylene production in bananas, modulating the expression of key ethylene-biosynthesis genes, and ultimately successfully delay ripening.

By contrast, in non-climacteric fruits, the dominant drivers of quality loss are often linked to oxidative processes and the progressive depletion of health-promoting metabolites. Accordingly, non-climacteric “red fruits”, such as strawberry, grape, cherry, and raspberry, are rich in beneficial bioactive compounds, mainly polyphenols, that tend to degrade rapidly during the postharvest stage. For this reason, the use of ECs to preserve their freshness and bioactive components has received considerable attention [69,70].

Regardless of their climacteric or non-climacteric nature, fruit ripening is regulated by complex physiological and biochemical mechanisms involving multiple metabolic signals and regulators, including organic acids and Reactive Oxygen Species (ROS) [71,72]. ROS metabolism plays a crucial role during the postharvest storage phase, where it exerts a dual effect. On the one hand, excessive accumulation can induce oxidative stress, triggering deterioration pathways. On the other hand, controlled ROS production contributes to defense response against pathogens [72]. Therefore, ECs capable of modulating ROS metabolism during postharvest storage are particularly relevant, as they can reduce oxidative stress and enhance the fruit’s endogenous antioxidant defenses. For instance, Guan et al. [73] used an EC formulated with montmorillonite, CH, and whey protein isolate to improve the postharvest quality of sweet potatoes. This coating was shown to upregulate the expression of several ROS metabolism-related genes, resulting in lower superoxide levels. In another study, Gull et al. [74] found that xanthan gum-based ECs effectively preserved the postharvest quality of guava fruits. In particular, the coatings were able to increase the activities of ROS-scavenging enzymes, improving the fruit’s oxidative stress response.

Infections caused by bacteria and fungi, which can originate either from latent infections or from wound injuries, represent another major threat to the food production chain. Considerable efforts have therefore been devoted to developing and assessing effective and sustainable EC-based strategies to control postharvest diseases and ensure food safety [75]. In this context, CH represents a particularly promising candidate, since in addition to its suitability as a substrate for EC formulation, it displays well-documented antifungal activity. Specifically, CH permeabilizes fungal plasma membranes, inducing the accumulation of intracellular ROS, ultimately compromising fungal viability. Furthermore, its action can interfere with key fungal cell wall components modulating the activity of enzymes involved in pathogenicity [76].

Another relevant advantage of ECs is their ability to incorporate antimicrobial agents, thereby further enhancing their protective properties [43]. Among these additives, bioactive peptides are particularly promising. Many peptides exert antimicrobial activity by electrostatically interacting with microbial cell membranes, which can lead to membrane destabilization and permeabilization [77]. For instance, Khedri et al. [78] incorporated casein phosphopeptides (CPPs) into gelatin-based ECs, obtaining improved mechanical properties, enhanced antioxidant capacity, and significant antimicrobial activity. Interestingly, the addition of CPPs was also shown to delay lipid oxidation that may contribute to the preservation of sensory and nutritional attributes during storage [79].

ECs supplemented with antimicrobial agents can further enhance their intrinsic antifungal activity and help control necrotrophic fungi, such as *Fusarium* spp. or *Botrytis cinerea* [80,81]. Safari et al. [82] showed that, in tomato, coatings enriched with CaCl_2_ or vanillin were able to significantly inhibit the mycelial growth of *Fusarium oxysporum*, one of the most aggressive postharvest pathogens affecting this crop. Strawberries are highly susceptible to rot caused by several fungal pathogens, with *B. cinerea* being the most prevalent; accordingly, a chitosan-based coating has recently been developed to extend shelf-life by reducing fungal growth [83].

Finally, Perez-Vazquez et al. [84] also emphasize that, among the attributes defining fruit and vegetable quality, functional formulations play an important role in maintaining the sensory properties of fresh products.

**Figure 2:**
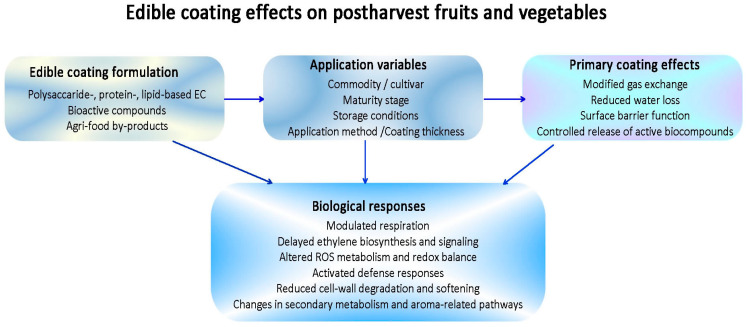
Schematic overview of the main factors and biological responses involved in edible coating effects on postharvest fruits and vegetables.

Figure 2 summarizes how ECs, including polysaccharide-, protein-, and lipid-based components, as well as coatings enriched with bioactive compounds or derived from agri-food by-products, may influence postharvest performance. It highlights how EC effectiveness is strongly influenced not only by coating composition, but also by product-related, application-related, and storage-related variables, which jointly determine barrier properties, coating–surface interactions, and the resulting physiological responses. After application, ECs can modify gas exchange, reduce water loss, act as surface barriers against pathogens, and regulate the release of active compounds. These primary effects may in turn modulate ripening- and senescence-related processes, redox balance, defense responses, cell wall metabolism, secondary metabolism, and aroma-related pathways, ultimately affecting fruit and vegetable quality, storability, and shelf-life.

Overall, ECs represent a valuable strategy for the postharvest management of intact fruits and vegetables as they create a protective barrier that modulates gas and moisture exchange, slows down physiological processes such as respiration and ripening [85].

## 5. Conventional and Omics-Based Approaches to Study the Effects of Edible Coatings

Traditionally, the effects of ECs on the postharvest quality of fruits and vegetables have been assessed using a wide range of conventional analytical techniques, primarily focused on physicochemical and microbiological parameters directly associated with product preservation during storage. The most commonly used indicators of coating performance include weight loss, firmness, color variations, and texture changes, which are generally employed as proxies of the ripening process [86,87]. Conventional approaches also rely on targeted biochemical assays to evaluate treatment-induced changes in specific metabolite classes, typically those of nutritional relevance and/or closely associated with ripening. Common measurements encompass TSS, titratable acidity (TA), total phenolics, flavonoids, carotenoids and anthocyanins, antioxidant capacity, as well as the quantification of individual metabolites of interest, with AA being among the most commonly assessed [88,89]. A complementary set of analyses includes enzymatic activity assays targeting enzymes known to influence product quality, most notably those involved in browning and visual appearance (PPO, POD) [90], antioxidant defense (SOD, CAT, APX) [73], and cell wall degradation associated with softening and texture loss (PG, PME) [91]. Microbiological assays are also routinely employed to evaluate the ability of ECs to inhibit the growth of undesirable microorganisms, including pathogenic fungi or molds [83].

Although these measurements provide valuable information on the coating’s ability to preserve quality over time, they offer only a limited view of the underlying metabolic changes induced by EC treatments. By capturing mainly broad phenotypic or chemical outcomes rather than the molecular processes driving them, conventional assays cannot fully elucidate how coatings modulate pathways involved in ripening, stress response, or quality deterioration. This limitation underscores the need for more advanced methods capable of probing EC effects at the molecular level.

In recent years, omics technologies have emerged as powerful tools to elucidate the complex biological processes underlying fruit and vegetable responses to ECs. These advanced analytical approaches provide a comprehensive view of how coatings influence postharvest physiology, quality attributes, and metabolic regulation, therefore representing a potential option for studying the response of fruits and vegetables to EC treatments. Unlike conventional assays that quantify a limited number of endpoints, omics approaches enable the simultaneous monitoring of thousands of molecular features, thereby investigating and clarifying coating–cuticle interactions, and capturing coating-induced reprogramming of ripening, senescence, and defense pathways.

Among the omics disciplines, transcriptomics has been applied to study EC-induced effects in fruits and vegetables. By employing RNA sequencing (RNA-Seq) or quantitative real-time PCR (qRT-PCR) workflows coupled with gene ontology (GO) enrichment analyses, gene expression analyses can identify differentially expressed genes (DEGs) associated with key metabolic and regulatory pathways, including those involved in cell wall remodeling, flavonoid biosynthesis, stress response, and pathogen defense [92].

Metabolomics has also been applied in EC research, and, compared with transcriptomic analyses, it provides a more direct readout of biochemical changes. By capturing the metabolic consequences of gene expression and other regulatory layers, metabolomics supports a more mechanistic understanding of how fruits and vegetables adjust their metabolic state in response to coating treatments. Accordingly, metabolomics is a powerful approach for the detection and quantification of metabolites that reflect the final outcome of multi-level cellular regulation [93,94]. In particular, metabolomic profiling can reveal how ECs reshape downstream metabolite levels and biochemical composition during storage.

In the following sections, we first summarize conventional analytical approaches used to evaluate sustainable ECs, including targeted metabolite analyses, and then review omics-based evidence, with a particular focus on transcriptomic, metabolomic, and integrated multi-omics investigations.

## 6. Conventional Studies on Coated Fruits and Vegetables

To date, most studies evaluating the effects of EC treatments on postharvest fruits and vegetables have relied on targeted metabolite analyses, together with physicochemical and analytical assays. In this section, we focused on recent studies investigating highly perishable fruits and vegetables treated with ECs, including those enriched with bioactive compounds derived from food industry by-products. We discuss representative cases involving figs, berries, peaches, and tomatoes, species particularly prone to postharvest deterioration and therefore ideal models to assess the effectiveness of EC-based preservation strategies. We then extended the analysis to other fruit categories, such as citrus and tropical fruits, and to selected vegetables, including potatoes. Finally, we considered recent studies on minimally processed fresh-cut fruits and leafy vegetables.

### 6.1. Highly Perishable Fruits

Figs are among the most perishable fruits, being highly susceptible to texture softening and skin cracking during the postharvest stage. In a study by Moccia et al. [95], the effects of an Active Edible Coating (AEC) were evaluated in order to extend the shelf-life while maintaining figs’ nutraceutical properties. Notably, figs treated with a polysaccharide-based edible mixture composed of sodium alginate, PE extracted from citrus peel, and Olive Leaf Extract (OLE) showed superior retention of nutritional and bioactive traits during storage. High-Performance Liquid Chromatography (HPLC) analysis confirmed higher retention of total flavonoids and other bioactive metabolites, underscoring the effectiveness of AECs in maintaining fig quality over time.

Strawberries were studied by Van et al. [96], who investigated the antifungal activity against *B. cinerea* of an EC composed of a mixture of CMC, Cellulose Nanofibers (CNF), and different concentrations of Mandarin Oil (MO) obtained from citrus processing by-products. MO, which was characterized by GC–MS/MS analysis, revealed a complex volatile profile dominated by β-pinene, limonene, and methyl palmitate, which are known to contribute to the antimicrobial and antioxidant properties of this citrus essential oil. Accordingly, incorporating MO exerted a strong antifungal effect against *B. cinerea.*

In addition to MO-based formulations, other essential oils have also been investigated as active components in ECs for strawberry preservation. Similarly, De Bruno et al. [89] evaluated the effects of a Gum Arabic (GA) coating supplemented with natural antioxidant extracts obtained from bergamot, pomace, and bergamot essential oil on strawberries during refrigerated storage. Targeted HPLC analysis highlighted the potential of these functional additives in EC formulation.

Gum-based EC effects have also been evaluated for other berry species. In a study conducted by Morodi et al. [90], different concentrations of GA were applied to red raspberries prior to drying. Alongside the assessment of several physicochemical and bioactive parameters, targeted LC–MS profiling of five individual anthocyanins revealed cyanidin dihexoside as the dominant pigment. The results indicated that GA pretreatment significantly improved several key quality attributes of dried raspberries, although this benefit was accompanied by a reduction in total anthocyanin, especially at higher gum concentrations. Changes in blueberry fruit quality were investigated by Liu et al. [97] through the application of two preservation strategies: a postharvest heat-shock treatment and a preharvest thymol-based EC application. GC–MS revealed that both treatments were able to preserve the aroma of fresh blueberries. While heat-shock treatments also helped maintain fruit quality at optimal temperatures, their positive effects were less pronounced than those of the EC. Interestingly, their combined application produced a synergistic effect, extending the shelf-life of blueberries by 7–14 days compared with the coating alone.

Torres-León et al. [98] successfully developed an active, biodegradable EC enriched with extracts from mango by-products to improve the postharvest preservation of peaches. In particular, the treatment led to an increase in TPC and a reduction in respiratory products, including CO_2_ and ethylene, suggesting that mango by-products can be effectively utilized to produce low-cost, biodegradable, and active packaging materials for fruit preservation.

Tomatoes represent another major target of postharvest preservation studies, owing to their high commercial value and pronounced susceptibility to quality deterioration, particularly fungal diseases, during storage and distribution. In a study, Poovai et al. [99] used a Hydroxypropyl Methylcellulose (HPMC)-based EC supplemented with piper betel leaf EO nanoemulsion as an antimicrobial bioactive compound, showing that the application of the coating significantly improved tomato resistance and quality during storage.

### 6.2. Other Fruits and Vegetables

The application of ECs on citrus fruits has been widely investigated, with mandarins emerging as the most extensively studied species.

In this context, the main underlying concept is the development of multifunctional ECs capable of providing, at the same time, an effective barrier effect and preserving the nutritional and organoleptic quality of fruits over time. Jurić et al. [100] developed coatings based on the Layer-By-Layer (LBL) technique, using both CH films and formulations incorporating different polyelectrolyte complexes derived from food by-products. These systems proved effective in maintaining physiological quality and protecting the nutraceutical value of mandarins during storage. More recently, Liguori et al. [101] reported similar outcomes by adopting a gellan gum-based coating enriched with Oregano Essential Oil (OEO) for the “Tardivo di Ciaculli” mandarin, a high-value cultivar of high commercial relevance.

Notably, complementary microbiological analyses demonstrated that ECs incorporating OEO significantly reduced mold viability, highlighting their potential for postharvest control in mandarins. In line with these findings, Soto-Muñoz et al. [102] demonstrated the effectiveness of ECs based on pregelatinized potato starch and glyceryl monostearate, either alone or supplemented with antimicrobial agents, in controlling sour rot, one of the major postharvest diseases affecting mandarins. Within the framework of postharvest fungal disease control in citrus fruits, Alvarez et al. [103] evaluated different antifungal agents for the formulation of PE–beeswax ECs aimed at controlling green mold caused by *Penicillium digitatum* in *Citrus sinensis* cv. ‘Valencia’ oranges. Among the tested compounds, the most promising results were obtained with *Commiphora myrrha*, geraniol, and eugenol, the latter being identified as the most suitable candidate for potential commercial application. On the same orange cultivar, Saberi et al. [104] investigated different composite ECs based on pea starch and guar gum, either blended with lipid mixtures or applied through an LBL approach.

Overall, these studies suggested that novel ECs could serve as a sustainable alternative to conventional commercial waxes, effectively maintaining storability and quality, not only in citrus fruits but also potentially in other fresh produce.

Another important class of products that has been extensively investigated in the development of innovative ECs is tropical fruits. This growing interest is largely driven by the need to ensure optimal postharvest preservation, given the long transportation times that many of these commodities must endure before reaching international markets, as well as by their high commercial value. Among the most extensively studied tropical fruits, mango stands out as a key model commodity. Ma et al. [105] applied a shellac and Tannic Acid (TA)-based coating, achieving a significant extension of shelf-life. Beyond this crucial aspect for ensuring successful distribution to international markets, the coating also proved effective in preserving fruit quality over time. Examples reported in the literature in which ECs have proven to be a valid alternative to conventional treatments are applied in apples [106], bell peppers [87], and potatoes [87,107].

### 6.3. Fresh-Cut Fruits

Fresh-cut fruits have gained remarkable popularity in recent years due to their convenience, but they remain highly perishable, being prone to rapid deterioration, which compromises their quality and shortens shelf-life [108]. Indeed, the fresh-cut processing triggers a series of physiological and biochemical events that lead to alterations in the sensory and nutritional quality of fruits [109]. The development of technologies capable of extending the shelf-life of these products and, more broadly, facilitating their postharvest management is, therefore, of particular relevance.

In this context, one of the main application targets, according to our review, appears to be melon, a fruit that is unsurprisingly often commercialized in fresh-cut form. Poverenov et al. [110] developed a CH-based coating exploiting by-products from the mushroom industry for the preservation of fresh-cut melon. When applied to fresh-cut melons, these fungal CH-based coatings were found not only to maintain fruit firmness and prevent off-flavors but also to significantly reduce bacterial growth. The valorization of food-industry by-products is also a central theme in the study by Cice et al. [111], who focused on the development of an AEC, applied through an LBL technique, based on sodium alginate and cedar mucilage. In this case, the application of the coating to fresh-cut melon provided highly promising results, highlighting not only the strong potential of ECs for postharvest preservation but also their role in promoting the sustainable use of food-industry by-products.

Another fruit that is frequently commercialized in fresh-cut form is the apple. In this case, the development of effective preservation strategies is even more critical, as apples are highly prone to rapid enzymatic browning after cutting and particularly susceptible to microbial infections. Among the most extensively studied coatings for fresh-cut apples are AECs, such as the AL-based formulation developed by Sarengaowa et al. [112], who investigated the incorporation of thyme oil, showing its ability to modulate enzymatic browning effects.

### 6.4. Minimally Processed Fresh-Cut Leafy Vegetables

Minimally processed fresh-cut leafy vegetables are among the most popular products in the food industry, yet they are also among the most susceptible to premature decay. Indeed, the development of new strategies to extend their shelf-life, such as the application of ECs, has become a major focus in current postharvest research.

Among the fastest processes that take place after cutting and lead to a rapid deterioration of both nutritional and sensory quality are enzymatic browning and oxidative senescence. Consequently, many studies in the field of ECs have focused on the development of strategies aimed at preserving bioactive pigments, such as carotenoids and chlorophylls, as well as phenolic compounds, thereby maintaining overall visual and nutritional quality during storage. Among the studies addressing this objective, Li et al. [113] investigated the effects of different EC formulations on the preservation of lettuce under refrigerated conditions, identifying CH-based coatings as those providing the most satisfactory results. More recently, Cofelice et al. [114] focused on ready-to-eat Salanova lettuce, a cultivar characterized by a particularly short shelf-life. In this study, several ECs supplemented with lemongrass essential oil were tested, with AL-based coatings emerging as the most effective in preserving bioactive pigments and phenolic compounds. Table 1 summarizes the above-discussed conventional studies.

## 7. Omics Studies on Coated Fruits and Vegetables

Omics approaches move beyond endpoint quality traits by enabling a systems-level view of how coatings influence ripening, stress physiology, and defense responses. However, to date, only a limited number of omics studies have investigated the effects of ECs on fruits and vegetables.

Here, we summarize transcriptomic and metabolomic studies, as well as multi-omics research on EC applications. Consistent with conventional studies, the focus is on fruit and vegetable species that are particularly susceptible to postharvest deterioration.

### 7.1. Transcriptomic Studies

Among the omics disciplines, transcriptomics has been by far the most widely applied for studying EC-induced effects in fruits and vegetables. Its widespread use likely reflects its relative accessibility: transcriptomics does not require highly specialized analytical platforms, benefits from standardized workflows, and relies on well-established bioinformatic pipelines.

#### 7.1.1. Highly Perishable Fruits

The banana is a climacteric fruit whose ripening process is strongly promoted by ethylene. This physiological feature results in rapid ripening and consequently a relatively short shelf-life. The application of a CH-based EC to extend banana shelf-life and its effects on the expression levels of genes belonging to the ACO family were investigated by Yamamoto et al. [68]. The results showed that ethylene output decreased despite ACO upregulation, consistent with O_2_ limitation at the enzyme level [25,115]. These findings provide insights into the molecular mechanisms underlying fruit ripening regulation by CH coatings and can support the development of targeted postharvest preservation strategies for bananas.

Subsequently, a transcriptomic approach showed that treatments with CH induce pathogen resistance in tomato [116]. In this study, the enhanced resistance to *B. cinerea* was associated with callose deposition and jasmonic acid accumulation. In addition, it was demonstrated that CH also primes the expression of specific defense-related genes, including *ACRE75* and *ACRE180*, which were functionally validated as positive regulators of resistance to *B. cinerea*. A targeted metabolomic analysis was also performed, and quantitative profiling of plant hormones was carried out using HPLC–MS techniques. Overall, the study provides valuable molecular insights into how CH enhances disease resistance, offering potential strategies to protect Solanaceae crops against *B. cinerea*.

Transcriptomic analyses have also been performed to investigate “natural” coatings, i.e., those already inherent to the fruit, such as the waxy bloom of blueberry. Most blueberry cultivars selected for the fresh market display an appealing light-blue coating (“bloom”), which results from the presence of a thick, visible layer of epicuticular wax. This waxy layer also serves as a natural defense against fruit desiccation and deterioration. In the study by Qi et al. [117], the aim was to identify genes associated with this protective waxy coating using two unique germplasm populations. RNA-Seq analysis identified a candidate gene, *FatB*, encoding an acyl-[acyl-carrier-protein] hydrolase, whose expression level was strongly correlated with segregation of the waxy trait. Specifically, *FatB* was expressed at more than fivefold higher levels in waxy accessions than in non-waxy ones across both populations analyzed. Overall, this study provided new insights into the molecular basis of wax biosynthesis in blueberry, contributing to a better understanding of cuticular lipid metabolism and fruit surface traits in this species.

#### 7.1.2. Other Fruits and Vegetables

Among the research areas in which transcriptomic approaches have been most extensively applied to the study of ECs, the investigation of host responses to fungal infections represents one of the most prominent. In the field of vegetables, potato is by far the most studied crop, being one of the most widely cultivated commodities worldwide and particularly susceptible to infections by *Alternaria tenuissima*, which can result in substantial postharvest losses.

Liu et al. [118] investigated the ability of CH-based coatings to counteract fungal growth in potato tubers. In addition to conventional microbiological and biochemical assays, the authors performed targeted gene expression analyses of defense-related enzymes using qPCR. Their results indicated that the reduced fungal growth observed in CH-treated samples could be attributed not only to a physical barrier effect but also to the activation of host defense mechanisms through the upregulation of defense-related genes, such as catalase, peroxidase, polyphenol oxidase, chitinase, and β-1,3-glucanase, as well as an increase in flavonoids and lignin levels, controlling tuber rot infection.

This hypothesis was further strengthened by the study of Ren et al. [119], in which CH-based coatings were applied to potatoes to control another major postharvest disease, Fusarium dry rot. In this case, an untargeted transcriptomic analysis based on RNA-Seq was conducted, providing a more comprehensive overview of coating-induced changes in gene expression. Approximately 5000 DEGs were identified, mainly associated with protein metabolism, stress tolerance, and cellular structure maintenance. Taken together, these studies suggest that CH-based coatings act both as direct antifungal agents and as elicitors of host defense responses, confirming their strong potential as natural fungicides for the postharvest control of potato diseases.

Transcriptomic approaches have also been successfully applied to several fruit species, again providing valuable insights into the mechanisms underlying the enhanced resistance to fungal infections induced by ECs. CH-based coatings emerge as the most extensively investigated systems, having been studied through targeted gene expression analyses using qPCR, as in the work of Zheng et al. [120], who explored the EC effects on kiwifruit. The ability of CH to enhance resistance against the postharvest gray mold (*B. cinerea*) and blue mold (*Penicillium expansum*) in kiwifruit was investigated, and the activity and gene expression of key antioxidant enzymes, as well as the TPC, were evaluated. The results showed that CH treatment upregulated the expression of APX, CAT, and SOD genes, suggesting that CH can activate the host antioxidant defense system, including the induction of antioxidant enzymes, thereby enhancing resistance against pathogen infection.

Avocado commercialization is often limited by severe postharvest losses caused by *Colletotrichum* spp., the causal agents of anthracnose. The RNA-Seq study by Xoca-Orozco et al. [121] aimed to identify genes differentially regulated by CH in the avocado–CH–*Colletotrichum* interaction system. Gene expression and GO analyses revealed a large number of DEGs involved in CH-dependent metabolic and defense processes, including those related to responses to bacterial and fungal pathogens and systemic acquired resistance (SAR). The results confirmed that CH exerts antifungal effects and acts as a direct elicitor of plant defense mechanisms, thereby enhancing resistance responses and broadening our understanding of the molecular pathways activated in fruits treated with this type of EC.

More recently, Hira et al. [122] investigated the molecular mechanisms potentially involved in shelf-life extension of the ‘Kosui’ Japanese pear (*Pyrus pyrifolia* Nakai) through the application of different ECs, including LBL applications. In this study, a global transcriptomic analysis based on RNA-Seq was performed to compare the effects of different coatings, providing particularly insightful results and demonstrating that distinct coating formulations can exert their protective effects through different cellular mechanisms. One of the most interesting outcomes of this work, which clearly highlights the potential of omics-based approaches for the study of ECs, is the evidence that specific coatings may be especially suited for the preservation of particular products depending on the cellular pathways they are able to modulate.

In the same year, a study on pear and apple [123] used RNA-Seq and qRT-PCR to explore molecular changes in these fruits after wax treatment. The results suggested that wax coating inhibits fruit ripening by affecting ethylene biosynthesis and signal transduction, chlorophyll metabolism, and carotenoid biosynthesis pathways, and that waxing also suppresses endogenous wax production. Overall, these findings provide new insights into how wax coatings delay fruit ripening.

### 7.2. Metabolomic Studies

As a relatively young field, metabolomics is typically performed on techniques such as gas chromatography–mass spectrometry (GC–MS) and liquid chromatography–mass spectrometry (LC–MS), platforms capable of capturing a broad spectrum of cellular end products. Despite their high potential to reveal mechanistic insights into EC-induced responses, the technical complexity and resource requirements of metabolomics have so far limited its application in postharvest coating research.

#### 7.2.1. Highly Perishable Fruits

In a study by Allegra et al. [124], the postharvest metabolic response of figs treated with a mucilage extract from *Opuntia ficus-indica* was investigated using an untargeted metabolomic approach based on GC-MS. The results demonstrated that EC application significantly prevented the loss of amino acids during cold storage and led to an increase in carbohydrates and other key metabolites, including β-sitosterol, glycerol, and uracil. Multivariate analyses also revealed clear metabolic differentiation between coated and uncoated fruits, highlighting the substantial impact of the treatment during postharvest storage and emphasizing its beneficial effects in preserving fruit quality. Overall, this study is noteworthy not only for the results obtained in this specific experimental context but also for effectively illustrating the potential of untargeted metabolomics approaches in the evaluation of postharvest preservation strategies, particularly for identifying pathway-related metabolic changes associated with coating treatments.

Another example of the application of metabolomics for the in-depth analysis of ECs is provided by the work of Yan et al. [70]. In this study, two different coating strategies were compared to evaluate their effectiveness in preserving postharvest strawberry quality. Fruits were treated either with a simple CH coating or with an LBL method, obtained by the alternating electrostatic deposition of CMC and CH. Results revealed that the LBL EC was markedly more effective than single-layer CH in inhibiting firmness loss and maintaining aroma-related volatile compounds. However, after eight days of storage, Layer-By-Layer -treated ECs displayed a stronger reduction in primary metabolites (involved in amino acids, carbohydrate, and fatty acids metabolism), as well as in secondary metabolites, such as those involved in carotenoid, phenylpropanoid, flavonoid, and terpenoid pathways.

More recently, Parijadi et al. [125] focused on a GC–MS-based analysis of volatile metabolite profiling during banana ripening. In this study, two postharvest preservation strategies were compared: one based on a CH-based EC and the other on cold storage. Interestingly, both approaches proved effective in delaying the ripening process, albeit through alterations in distinct metabolic pathways. This study highlights an additional and still underexplored potential of untargeted metabolomics in the field of ECs, namely its ability to elucidate how different coating formulations or preservation strategies can differentially impact metabolic pathways underlying fruit ripening and senescence.

#### 7.2.2. Other Fruits and Vegetables

Beyond the most critical cases of highly perishable fruits, metabolomics-based approaches have also been applied to a broader range of fruit and vegetable commodities. In their study, Fonseca et al. [126] investigated the effects of PE-based ECs on the volatilome in the context of preserving ‘Rocha’ pears during cold storage. Notably, the authors employed a comprehensive two-dimensional gas chromatography platform coupled with mass spectrometry (GC × GC–MS), which enabled an expanded coverage of volatile metabolites. This enhanced analytical resolution allowed them to demonstrate that the combination of dynamic controlled atmosphere and PE ECs reduced the emission of ripening-associated volatiles, a response that may contribute to extending the storage life of ‘Rocha’ pears.

Modulation of the volatilome following coating application was also a major focus of Ikram et al. [127], who used conventional one-dimensional GC–MS to analyze fresh-cut pineapple treated with CH-based coatings, with or without melezitose. The addition of melezitose affected cell wall metabolism and showed potential to prolong the shelf-life of fresh-cut pineapple, providing a basis for further postharvest studies on whole pineapple fruit.

More recently, CH-based coatings have also been explored by Cao et al. [128] in the context of kiwifruit preservation. In this work, alongside conventional CH formulations, innovative CH–silica nanoparticle coatings were developed and evaluated, showing superior preservation performance. In both studies, metabolomic analyses proved instrumental in clarifying which metabolic features were most affected by the coating treatments, providing insights into the biochemical basis of their preservative effects.

An LC–MS-based metabolomics approach was instead adopted by Wang and Gong [129] to explore a strategy aimed at delaying senescence and preserving the quality of snap bean pods. In this study, a CMC/starch-based photodynamic film was evaluated both under dark conditions and following photodynamic activation. Among the approximately 1800 metabolites detected, about 450 were found to be significantly altered in samples subjected to photodynamic treatment. Notably, the photodynamic packaging proved particularly effective in slowing the deterioration of several classes of compounds, including phenolic acids, flavonoids, and lipids, highlighting the added value of combining innovative coating technologies with metabolomics to elucidate their mode of action.

### 7.3. Combined Transcriptomic and Metabolomic Studies

The combination of transcriptomics and metabolomics is a particularly powerful approach that can help identify key factors and stress-related proteins in crops, allowing researchers to gain precious insight into the regulatory mechanisms of key metabolites at the molecular level and improve nutritional and qualitative traits of postharvest fruits and vegetables [130].

Also in multi-omics studies, CH-based ECs have been the most extensively investigated.

In the study by Zhang Z. et al. [131], the combination of metabolomics and transcriptomics was instead crucial to suggest differences in the response of two grape varieties to a pathogen following the application of the same CH-based coating. In particular, the two selected varieties are characterized by different susceptibility to *B. cinerea.* The analysis revealed that, in both varieties, CH treatment was able to activate defense mechanisms against the pathogen, although to different extents, as expected. However, at the metabolomic level, much more substantial differences emerged, highlighting how the two varieties accumulated different metabolites (mainly catechins, resveratrol, and other polyphenols), which suggests the adoption of distinct metabolic strategies. The combination of transcriptomics and metabolomics, therefore, provides a broader perspective than that obtainable from single-omics approaches when investigating the effects of ECs, and it is highly desirable that this strategy be applied more extensively in future studies in this field.

Another clear example of the value of integrated metabolomic and transcriptomic analyses in EC research comes from a recent study by Yang et al. [132] on postharvest kiwifruit coated with CH. Transcriptomic analyses revealed several DEGs, including a downregulation of genes potentially involved in cell wall modification and three starch degradation-related genes, which can be correlated with fruit softening and loss of firmness. At the same time, an upregulation of genes associated with flavonoid biosynthesis was observed, which can be interpreted as an activation of fruit defense mechanisms. Metabolomic analyses confirmed that these DEGs effectively resulted in changes in the final metabolites, demonstrating that the observed transcriptional variations were coherent and biologically relevant.

Subsequently, to elucidate the regulatory mechanisms through which carboxymethyl chitosan (CMCS)-based ECs mitigate postharvest softening in okra, the authors performed an integrated analysis combining physiological measurements with transcriptomic and metabolomic profiling [133]. Overall, CMCS treatment delayed visible quality decline and reduced chlorophyll breakdown. In addition, CMCS suppressed the activities of key pectin-degrading enzymes, such as PE and PG. Pathway-level analyses related to texture and quality further indicated that CMCS downregulated genes associated with membrane lipid catabolism and helped maintain redox homeostasis by modulating the expression of antioxidant-related genes. Notably, CMCS reprogrammed phytohormone dynamics by reducing ethylene biosynthesis while increasing ABA-related expression. Finally, combined transcriptomic and metabolomic integration analysis revealed a marked activation of the flavonoid pathway, with both higher levels of flavonoid metabolites and increased expression of flavonoid biosynthetic genes.

Peach gum-based coating was also the subject of multi-omics studies. In Zhang L. et al. [134], the potential of this coating for postharvest preservation of peaches during cold storage was investigated by using transcriptomics and non-targeted metabolite profiling techniques. The treatment inhibited ethylene production, maintained firmness, and reduced weight loss, while largely preserving the levels of major sugars and organic acids (citric, malic, and quinic acids; glucose, fructose, and sucrose). Consistently, transcriptomics showed downregulation of genes involved in cell wall degradation/softening and suppression of cold storage-induced pathogenesis-related and senescence-associated genes. Overall, peach gum emerges as a promising natural EC for preserving peach quality during postharvest storage.

The previously discussed studies employing multi-omics approaches shed light on how ECs modulate physiological and molecular responses during postharvest storage and enable the identification of potential markers for evaluating coating performance and optimizing formulations. Table 2 summarizes the above-discussed transcriptomics, metabolomics, and combined metabolomics–transcriptomics studies.

## 8. Discussion, Concluding Remarks, and Future Perspectives

This review confirms that EC applications represent a rapidly expanding research field, increasingly explored by the scientific community in response to the need to reduce food waste, growing consumer demand for fresh and minimally processed products, and rising societal awareness of environmental sustainability.

Across the literature reviewed, EC effectiveness emerges from the interplay between physical barrier functions and biologically active effects. On the one hand, coatings modify the microenvironment at the fruit surface, altering gas exchange and water loss, which in turn impacts respiration, ethylene production, and softening-related processes. On the other hand, EC formulations and coatings enriched with essential oils or phenolic-rich extracts appear to elicit defense responses consistent with priming-like mechanisms reported at both transcript and metabolite levels.

Additionally, the discussed studies underline the strong commodity and context-dependence of EC outcomes. Climacteric fruits, such as banana, may benefit primarily from reduced oxygen availability and delayed ethylene-mediated ripening, whereas in other commodities the dominant effect may involve modulation of antioxidant capacity, phenylpropanoid metabolism, or microbial growth at the surface. This variability likely reflects differences in cuticle structure, endogenous hormone dynamics, and pathogen susceptibility, highlighting the importance of tailoring EC formulations to specific commodities, maturity stages, and storage conditions rather than assuming a one-size-fits-all solution.

The literature reviewed encompasses both conventional analytical and biochemical methods, including targeted metabolite analyses, and omics-based approaches, mainly focused on transcriptomics and metabolomics.

Conventional studies often prioritize immediate preservation effects rather than clarifying the molecular mechanisms governing diffusion and interactions between coating components and the fruit/vegetable matrix, including the cuticle and epidermal tissues, which ultimately shape mass transfer and biological responses during storage. Nevertheless, targeted metabolite studies provide quantitative, mechanism-oriented, and decision-relevant readouts that directly link coating properties to postharvest physiology and quality outcomes. For this reason, targeted metabolite studies have been given particular attention in the conventional section of this review.

In parallel, omics approaches are especially useful for investigating EC effects, as these treatments do not act solely as physical barriers but can also substantially modulate fruit and vegetable metabolism, thereby influencing the physiological, biochemical, and molecular processes that govern postharvest quality and shelf-life extension. In particular, the transcriptomic and metabolomic studies reviewed here demonstrate that coatings can modulate ripening- and stress-related processes, affecting hormone signaling, redox homeostasis, cell wall metabolism, secondary metabolism, and aroma-related pathways.

Within this framework, metabolomics is particularly valuable for identifying the pathways and metabolites that respond to coatings, including those related to respiration, ethylene/ACC metabolism, oxidative stress, phenylpropanoid and carotenoid metabolism, amino acid and organic acid metabolism, and volatile compound production. Overall, the reviewed studies show that metabolomics is especially useful for mapping coating-responsive pathways and uncovering unexpected metabolic signatures, whereas targeted metabolite analysis enables accurate quantification of key metabolites and hormones with high practical relevance.

Moreover, the available omics evidence indicates that integrating transcriptomics and metabolomics provides a more comprehensive understanding of EC treatment outcomes during postharvest storage. Combining these approaches enables the identification of coating-responsive metabolites and genes that influence ripening and other quality-related traits in fruits and vegetables. Accordingly, the multi-omics studies discussed here provide the most comprehensive view by linking regulatory changes to downstream metabolic outcomes, thereby supporting a more mechanistic interpretation of coating effects and enabling the identification of candidate molecular markers associated with coating effectiveness.

At the same time, this review highlights several important limitations in the current literature. A major challenge is the marked heterogeneity among studies, including differences in commodities, cultivars, maturity stages, coating formulations, application methods, storage conditions, and sampling strategies, which makes cross-study comparisons difficult. Another critical gap concerns the still-limited mechanistic understanding of how coating components interact with the fruit or vegetable surface, including the cuticle, epidermal tissues, and possibly the surface microbiota, and how these interactions ultimately influence mass transfer and biological responses during storage. In addition, although multi-omics studies are highly informative, they are still too scarce to establish a robust mechanistic framework across commodities and coating types, and experimental designs remain insufficiently standardized. More time-resolved studies are therefore needed to capture the dynamics of coating-induced changes, particularly in relation to respiration, ethylene-related pathways, oxidative balance, secondary metabolism, and defense responses. The adoption of harmonized analytical workflows would also improve reproducibility and facilitate cross-study comparisons.

Additionally, most available studies on EC-treated fruits and vegetables still rely on conventional approaches, whereas omics-based investigations remain comparatively scarce.

This represents a clear shortcoming in EC research, which the present review seeks to address by providing a more integrative perspective and an innovative framework for interpreting coating performance at both applied and molecular levels. In particular, this work fills an important gap in the EC literature by systematically examining transcriptomic, metabolomic, and multi-omics evidence, which has so far been addressed only fragmentarily and has not yet been comprehensively integrated into existing review papers. This broader perspective helps connect molecular-level responses with physiological and quality-related outcomes, thereby supporting a more mechanistic interpretation of coating performance.

**Figure 3:**
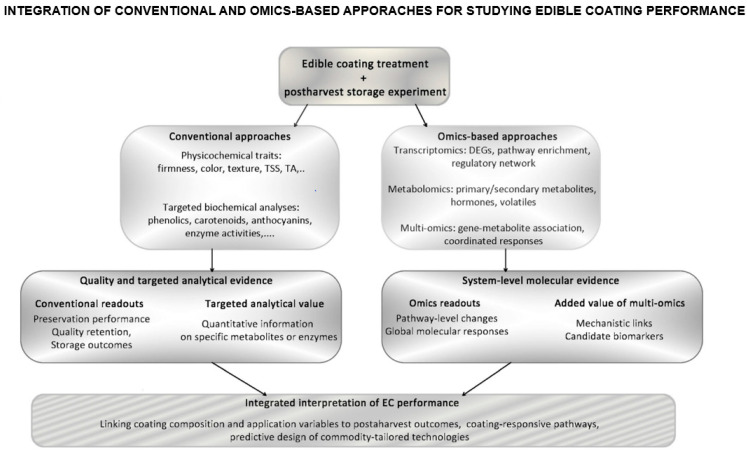
Schematic representation of the complementarity between conventional analytical methods and omics-based approaches in the study of EC effects and performance, highlighting their integration for a more mechanistic interpretation of postharvest responses.

From this perspective, the review also outlines possible future research strategies. In particular, it highlights how a wider adoption of standardized, time-resolved omics and multi-omics frameworks, integrated with conventional quality metrics, could be instrumental in developing more predictive and sustainable postharvest coating technologies. As illustrated in Figure 3, the integration of conventional quality assessments with transcriptomic, metabolomic, and multi-omics approaches may help connect postharvest outcomes with the underlying biological processes modulated by ECs.

Conventional analytical methods provide performance-oriented evidence on quality retention and storage outcomes, as well as targeted analytical measurements, whereas transcriptomics, metabolomics, and multi-omics integration reveal pathway-level and systems-level molecular responses. Their combined use may therefore support a more mechanistic interpretation of coating performance and a more predictive design of edible coating strategies.

Therefore, one of the major future challenges will be to move beyond largely descriptive evaluations of coating performance toward more mechanistic and predictive frameworks.

From an application-oriented perspective, future research should increasingly aim to connect molecular profiling with practical postharvest performance. In particular, combining conventional measurements with transcriptomics, metabolomics, and multi-omics integration may support the identification of candidate biomarkers associated with coating efficacy, product suitability, or storage stability. This would be especially valuable for moving from empirical coating development toward more evidence-based and commodity-tailored design.

Beyond the integration of conventional approaches with transcriptomic, metabolomic, and multi-omics tools, emerging technological solutions such as nanotechnology may further expand the design and functional potential of next-generation ECs. As discussed in this review, nanomaterials such as nanocellulose, nanoemulsions, and inorganic nanoparticles have been increasingly explored to enhance the mechanical strength, barrier properties, and antimicrobial activity of coating matrices. However, studies combining nano-enabled coatings with omics approaches are still scarce, and their interaction with plant tissues, as well as their potential influence on fruit physiology and metabolism, remain largely unexplored. In this context, omics approaches could provide valuable insights into the molecular responses triggered by nano-enabled coatings, allowing the identification of changes in gene expression, metabolic pathways, and stress-related processes during storage. Integrating nanotechnology with transcriptomic, metabolomic, and multi-omics analyses could therefore contribute to a more mechanistic understanding of coating–fruit interactions and support the rational design of more effective, commodity-tailored, and biologically compatible postharvest preservation strategies.

Additional challenges include formulation reproducibility, scalability, cost-effectiveness, and the maintenance of sensory acceptability, all of which are crucial for successful implementation in the agri-food sector.

Despite these challenges, these integrated perspectives are highly relevant to the agri-food industry, as they may support the development of more rational, effective, a commodity-tailored edible coating strategies for postharvest preservation, helping to improve product quality, reduce losses, and enhance sustainability along the supply chain.

On this basis, we hope that this review will serve as a useful starting point and conceptual framework for researchers seeking to apply omics approaches in this field.

## Figures and Tables

**Table 1:** Summary of recent conventional physicochemical and targeted analytical studies on coated fruits and vegetables.

Fruit Vegetable	EC Composition	Main Results	Targeted Metabolite Analysis Techniques	References
Fig	AEC including AL, PE from citrus peel and OLE	Extension of the shelf-life and preservation of nutritional properties	HPLC	[95]
Strawberry	GA, Bergamot EOs and pomace	Coated samples showed slower decay rates and good AA retention	HPLC	[89]
Strawberry	CMC, CNF and MO	MO addition into coatings helped control weight loss and showed relevant antifungal activity	GC-MS	[96]
Red raspberry	GA	GA coating improved AA content, TPC, TSS, and water activity, while reducing color and total antho-cyanin content	LC-MS	[90]
Blueberry	(Thymol/KGM)/LAG—(TKL)	The combination of (Thymol/KGM)/LAG—(TKL) treatments extends the shelf-life of blueberries compared to coating alone	GC-MS	[96]
Mandarin	LBL: AL/CH, locust bean gum/CH and HPMC/CH	The best results were observed for the LBL HPMC/CH coating	HPLC	[100]
Mandarin	Gellan gum, glycerol, calcium chloride, distilled water, and OEO	ECs effectively preserved quality, minimizing water and weight loss. EC incorporating OEO reduced mold viability	(SPME) GC-MS	[101]
Mandarin	PPS and GMS	The PPS-GMS-based coatings can significantly increase the content of ethanol and acetaldehyde	GC	[102]
Orange	PE-based ECs with EOs	ECs containing GE reduced disease incidence, while CN effectively reduced disease severity	GC-FID	[103]
Orange	PSGG and pea starch, PSGG and lipid mixture with oleic and shellac (PSGG–Sh), LBL	The incorporation of lipids into PSGG–Sh was the best treatment for coated ‘Valencia’ oranges	FID	[104]
Mango	Shellac and TA	TA–shellac extended shelf-life and improved overall fruit quality	GC-IMS	[105]
Peach	Mango peel and antioxidant extracts of seed kernel	By-products of mango may be suitable for the production of low-cost biodegradable active packaging	GC	[98]
Apple	Sucrose monoesters of fatty acid and ethanol	This kind of coating allowed reduced respiration rates in apple and improved the shelf-life	GC	[106]
Tomato	HPMC with piper betel leaf EO nanoemulsion	This kind of EC delayed changes in color, weight loss, and positively affected AA and lycopene content	GC-MS	[99]
Pepper	Gum tragacanth coating	The EC treatment led to lower weight loss and higher total carbohydrates and total carotenoids content	HPLC	[87]
Fresh-cut potato, fried potato	CMC, flaxseed mucilage, burdock extract	The antioxidant activity of fresh-cut potato, phenolics, and vitamin C were preserved. For fried potato, AEC prevented acrylamide formation	GC-FID	[107]
Fresh-cut melon	Mushroom industry wastage as source of antimicrobial CH	The coated melon exhibits a higher ester content, directly responsible for the typical fruit flavor	GC-MS	[110]
Fresh-cut melon	LBL: sodium alginate and cedar mucilage	LBL EC reduced weight loss and enhanced polyphenol, flavonoid, AA content and antioxidant activity	UV–VIS	[111]
Fresh-cut apple	AL-based EC containing thyme oil (EO)	AL-based EC containing thyme EO may be a promising approach to extend the shelf-life of fresh-cut apples	GC-MS	[112]
Fresh-cut lettuce	Polysaccharide-based EC (AL, carrageenan and CH)	CH coating showed the best effects on quality and was the most suitable to delay enzymatic browning and reduce oxidative damage	GC-MS	[113]
Ready-to-eat lettuce	Lemongrass EO within different polymers: AL, chia mucilage, and CH	AL coating proved to be the most effective in preserving bioactive pigments and phenolic compounds of lettuce	HPLC	[114]

**Table 2:** Summary of significant omics (transcriptomics and metabolomics) and multi-omics studies on coated fruits and vegetables.

Fruit Vegetable	EC Composition	Main Results	Technique	References
Banana	CH	ACO gene transcription was promoted, but the downstream process of the ethylene production could be inhibited	qRT-PCR	[68]
Tomato	CH	A transcriptomic analysis revealed that CH gene expression at early phases of the infection and is able to control *B. cinerea*	Microarrays HPLC-MS	[116]
Blueberry	Natural protective waxy coating	A candidate gene, *FatB*, encoding an acyl-[acyl-carrier-protein] hydrolase, was found. Its expression is closely related to waxy coating character segregation in the investigated populations	RNA-Seq	[117]
Potato	CH	CH induced the expression of defense-related genes, such as catalase, peroxidase, PPO, chitinase, and β-1,3-glucanase, controlling tuber rot infection caused by *A. tenuissima*	qRT-PCR	[118]
Potato	CH	Transcriptome analysis showed that DEGs were mostly related to protein metabolism, stress tolerance, and cell structure, suggesting that CH might be a promising natural fungicide against *F. oxysporum* infection	RNA-Seq	[119]
Kiwifruit	CH	CH induced ascorbate peroxidase, catalase, and superoxide dismutase gene expression. CH promotes resistance to gray disease and blue mold	qRT-PCR	[120]
Avocado	CH	A large number of DEGs involved in CH-dependent metabolic processes, including those preventing *Colletotrichum* infection spread, was identified	RNA-Seq	[121]
Pear	LBL: CH/AL-based EC	A downregulation of genes associated with fruit maturation and ethylene production was observed	RNA-Seq	[122]
Pear, apple	Wax-based EC	Wax-based EC inhibits fruit ripening by affecting ethylene biosynthesis and signal transduction, chlorophyll metabolism, and carotenoid pathways	RNA-Seq qRT-PCR	[123]
Strawberry	LBL: CMC and CH	LBL EC significantly inhibited firmness and aroma loss	GC-MS	[70]
Fig	*Opuntia ficus-indica* Mucilage	Reduction of the amino acid content loss and increase of the carbohydrates and other relevant metabolites, including beta-sitosterol, glycerol, and uracil	GC-MS	[124]
Banana	CH	After 11 days, CH-coated fruit showed a marked accumulation of 1-aminocyclopropane-1-carboxylic acid	GC-MS	[125]
Pear	PE-based	The combination of dynamic controlled atmosphere conditions with PE ECs reduced the emission of ripening-associated volatiles (esters and sesquiterpenes)	GC × GC ToFMS	[126]
Pineapple	CH with melezitose	This EC prolonged the shelf-life of fresh-cut pineapple. Results showed significant alteration of the primary metabolites: quinic acid, sucrose, and xylitol	GC-MS	[127]
Kiwifruit	CH and CH-silica nanoparticle (CHS-SiNPs)	A total of 32 compounds were identified as key aroma-active compounds, and most green leaf volatiles and methyl salicylate were upregulated in the CHS-SiNPs coating groups	HS-SPME GC–MS	[128]
Snap bean	CMC/starch-based photodynamic film (CCPF)	It was found that CCPF could delay the deterioration of the quality of snap bean pods	QQQ MS/MS	[129]
Grape	CH	A large number of secondary metabolites differed between the two examined grape varieties, such as catechin, epigallocatechin gallate, and resveratrol, which could explain their different susceptibility	RNA-Seq LC–MS/MS	[131]
Kiwifruit	CH	One cell wall modification and three starch degradation genes, one gene involved in flavonoid biosynthesis, and TFs, such as bZIP, IAA, bHLH, may be involved in controlling ripening and quality	RNA-Seq UPLC-ESI-MS/MS	[132]
Okra	CMCS	CMCS treatment delayed visible quality decline and reduced chlorophyll breakdown. It also suppressed the activities of key pectin-degrading enzymes	RNA-Seq LC-MS	[133]
Peach	Peach-gum coating	This treatment inhibited ethylene production, helped maintain firmness, and prevented weight loss	RNA-Seq GC–MS	[134]

## Data Availability

No new data were created or analyzed in this study.

## Abbreviations

The following abbreviations are used in this manuscript:

AA	Ascorbic Acids
AEC	Active Edible Coating
AL	Alginate
APX	Ascorbate Peroxidase
CH	Chitosan
CA	Citric Acids
CAT	Catalase
CMC	CarboxyMethyl Cellulose
DEGs	Differential Expressed Genes
EOs	Essential Oils
GA	Gum Arabic
GC	Gas Chromatograph
GC-IMS	GC-Ion-Mobility Spectrometry
GRAS	Generally Recognized As Safe
HPLC	High Performance Liquid Chromatography
LC-MS	Liquid Chromatography-Mass Spectrometry
LBL	Layer-By-Layer
MAP	Modified Atmosphere Packaging
MS	Mass Spectrometer
OLE	Olive Leaf Extract
OEO	Oregano Essential Oil
PAL	Phenyl-alanine Ammonia Lyase
PCR	Polymerase Chain reaction
PE	Pectin
PG	Polygalacturonase
POD	Phenol Peroxidase
PME	Pectin Methylesterase
PPO	Polyphenol Oxidase
RNA-Seq	RNA Sequencing
ROS	Reactive Oxygen Species
SOD	Superoxide Dismutase
TPC	Total Phenolic Content
TSS	Total Soluble Solids
VOCs	Volatile Organic Components
